# Inertial Sensor-Based Motion Tracking in Football with Movement Intensity Quantification

**DOI:** 10.3390/s20092527

**Published:** 2020-04-29

**Authors:** Erik Wilmes, Cornelis J. de Ruiter, Bram J. C. Bastiaansen, Jasper F. J. A. van Zon, Riemer J. K. Vegter, Michel S. Brink, Edwin A. Goedhart, Koen A. P. M. Lemmink, Geert J. P. Savelsbergh

**Affiliations:** 1 Amsterdam Movement Sciences, Department of Human Movement Sciences, Faculty of Behavioural and Movement Sciences, Vrije Universiteit Amsterdam, 1081BT Amsterdam, The Netherlands; c.j.de.ruiter@vu.nl (C.R.d.R.); jasperzon@hotmail.com (J.F.J.A.v.Z.); g.j.p.savelsbergh@vu.nl (G.J.P.S.); 2 Center for Human Movement Sciences, University Medical Center Groningen, University of Groningen, 9713AV Groningen, The Netherlands; b.j.c.bastiaansen@umcg.nl (B.J.C.B.); r.j.k.vegter@umcg.nl (R.J.K.V.); m.s.brink@umcg.nl (M.S.B.); k.a.p.m.lemmink@umcg.nl (K.A.P.M.L.); 3 FIFA Medical Centre of Excellence, Royal Netherlands Football Association, 3707HX Zeist, The Netherlands; edwin.goedhart@knvb.nl

**Keywords:** inertial measurement units, lower body kinematics, soccer, physical load, movement intensity, exercise

## Abstract

Inertial sensor-based measurements of lower body kinematics in football players may improve physical load estimates during training sessions and matches. However, the validity of inertial-based motion analysis systems is specific to both the type of movement and the intensity at which movements are executed. Importantly, such a system should be relatively simple, so it can easily be used in daily practice. This paper introduces an easy-to-use inertial-based motion analysis system and evaluates its validity using an optoelectronic motion analysis system as a gold standard. The system was validated in 11 football players for six different football specific movements that were executed at low, medium, and maximal intensity. Across all movements and intensities, the root mean square differences (means ± SD) for knee and hip flexion/extension angles were 5.3° ± 3.4° and 8.0° ± 3.5°, respectively, illustrating good validity with the gold standard. In addition, mean absolute flexion/extension angular velocities significantly differed between the three movement intensities. These results show the potential to use the inertial based motion analysis system in football practice to obtain lower body kinematics and to quantify movement intensity, which both may improve currently used physical load estimates of the players.

## 1. Introduction

The analysis of lower body kinematics of a football player throughout a training or match can become a useful tool to improve currently used physical load estimates. Presently, radio frequency-based local positioning measurement systems (LPM) and satellite-based global positioning systems (GPS), which measure a player’s position on the field continuously, are widely used to quantify physical load during practice and competition [1,2,3]. However, many explosive actions associated with high muscle loads, such as accelerations, decelerations, kicking, jumping, and side-cutting, do not necessarily involve large or fast global displacements [4,5,6]. Even LPM, which is considered to be more accurate compared to GPS-based systems, does not provide accurate estimations of instantaneous acceleration during explosive movements [7], while such movements place extensive mechanical loads on muscles, tendons, and joints. This is highly relevant because mechanical muscle loading is thought to be an important cause of muscle injuries in football, especially for muscles around the hip [3,8]. Consequently, a considerable amount of external training load may be missed using LPM or GPS systems only. Therefore, external training load estimates in football may be improved by inclusion of lower body kinematics, which also opens the possibility for the quantification of football specific actions, like ball kicking, cutting movements, or explosive short distance sprints. 

Movement kinematics have traditionally been obtained using optoelectronic motion analysis systems. However, these systems are restricted to a laboratory setting, have a limited measurement volume, and involve extensive start-up procedures, which make them unsuitable for use in daily football practice. Moreover, optoelectronic motion analysis requires a clear line-of-sight between cameras and markers, which may be obstructed by the ball or other players. Wearable inertial-based motion analysis systems have recently gained popularity because these systems allow for registration of movement kinematics without these limitations [9,10]. Inertial-based motion analysis systems consist of inertial magnetic measurement units (IMUs) attached to body segments. These sensors directly measure linear acceleration, angular velocity, and magnetic field strength in three orthogonal axes. The orientation of each IMU is obtained by combining these sensor readings in sensor fusion algorithms [11]. In combination with a biomechanical model, joint and body segment kinematics can be obtained [12]. A sensor-to-segment calibration needs to be performed to construct the biomechanical model. A variety of methods have been used to do so. However, most of these methods require external devices or take up a considerable amount of time [13]. To use an inertial-based motion analysis system to quantify lower body kinematics on a daily basis, sensor-to-segment calibration should be quick and easy to perform.

Inertial-based motion analysis systems have been applied successfully to estimate movement kinematics in sports such as marathon running [14] and swimming [15,16]. Such systems have also shown good agreement with gold standard optoelectronic systems in quantifying lower body kinematics during various football related activities studied in isolation, such as walking [17,18], running [13], and kicking [19]. However, the accuracy of inertial motion analysis systems depends on the type of movement and the intensity at which a movement is performed [10,20]. Moreover, soft tissue artefacts can be expected to increase in higher intensity movements. To the best of our knowledge, no studies have assessed the validity of an inertial based motion analysis system for a variety of common football specific movements, such as accelerating, decelerating, cutting movements, and turning. Moreover, the maximum running speed for which an inertial-based system has been validated is ~3.9 m/s [21], while running speeds above 5.5 m/s frequently occur during professional football matches [22]. Therefore, the validation of an inertial motion analysis system for football should include a wide variety of football-specific movements performed at high intensities.

The intensity of a movement may be estimated by measuring the angular velocity of the joints involved in a similar way to how exercise intensity is now determined from velocity measures obtained by LPM and GPS [1]. In sprinting, for example, joint angular patterns are shown to be relatively invariable across a range of speeds. As a consequence, the energy associated with the lower limbs is approximately proportional to the joint angular velocities of legs [23]. Therefore, movement intensities may be estimated by measuring joint angular velocities.

Considering the potential additional value of quantifying lower body kinematics to physical load estimates in daily football practice, this paper proposes a relatively simple inertial-based system. Especially, the sensor-to-segment calibration procedure is straightforward, fast, and does not require an experienced operator. The primary aim of the study was to assess the concurrent validity of the inertial-based system with an optoelectronic motion analysis system for a variety of football specific movements performed at submaximal and maximal intensities. Knee and hip flexion/extension angles and angular velocities were evaluated because most muscle injuries in football affect the muscles around the hip and knee [8]. The secondary aim of the study was to establish whether movement intensities can be distinguished based on joint flexion/extension (FE) angular velocities obtained by the inertial-based system. We hypothesized that the inertial motion analysis system would show good concurrent validity with the optoelectronic system. However, we expected larger differences between the systems for movements performed at maximal intensities. Moreover, we anticipated that we would be able to differentiate between movement intensities based on joint angular (flexion/extension) velocities.

## 2. Materials and Methods

### 2.1. System Setup

#### 2.1.1. System Hardware

The inertial motion analysis system consisted of five 9-DOF IMUs (MPU-9150, Invensense, San Jose, CA, USA). Each of these IMUs measured 3D acceleration, 3D angular velocity, and 3D magnetic field strength in a local coordinate frame attached to the sensor. Every sensor was embedded in a protective casing together with a battery and SD-card (total weight = 11 g), onto which the data was logged at a sample frequency of 500 Hz. This allowed for offline analysis after a measurement period. Before measurements, the sensors were placed in a small box, which was tapped on a table. This introduced a mechanical peak in the accelerometer signals, to which the sensors were time-synchronized [24]. 

**Figure 1:**
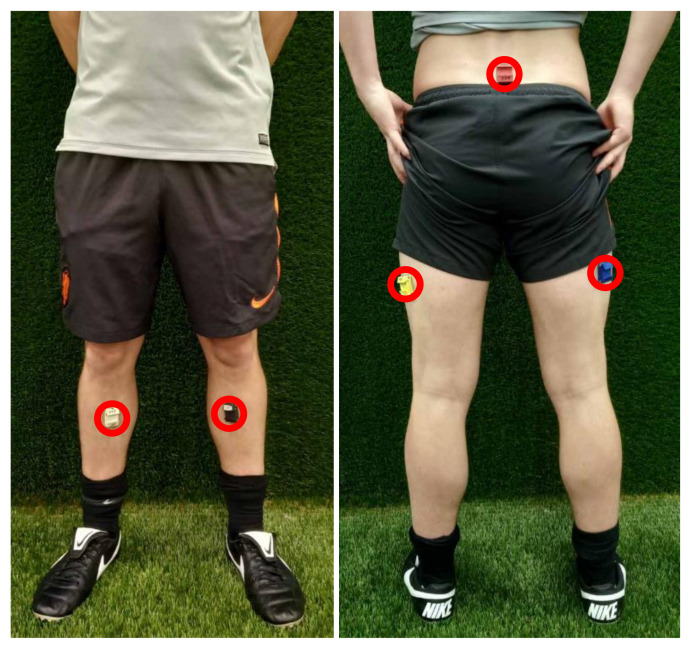
Sensor placement. The sensors were placed at the sacrum, iliotibial tracts, and shins. For clarity, the covering adhesive plaster was left out of the picture.

Subsequently, and as described in detail elsewhere [25], the sensors were placed on the pelvis, thighs, and shanks. They were rigidly attached to each body segment using pretape adhesion spray (Mueller Tuffner Pre-Tape Spray, Mueller sports medicine, Prairie du Sac, Wisconsin, United States of America) and double-sided adhesive tape (Begasoft-Airband, Bergmann GmbH & Co. KG, Laupheim, Germany). An adhesive plaster (Fixomull stretch, BSN Medical, Zeist, The Netherlands) was put over each IMU for extra fixation of the sensors to the skin. In an attempt to minimize soft tissue artefacts, the sensors were placed where underlying tissue was expected to show the least deformation. The pelvis sensor was placed at the sacrum, the thigh sensors were placed at the iliotibial tract, and the shank sensors were placed on the shin (Figure 1). Gyroscope measurements were low-pass filtered using a second-order zero-lag Butterworth filter with a cutoff frequency of 12 Hz to eliminate soft tissue artefacts. The orientation of each sensor throughout a measurement period was obtained using a gradient descent Madgwick algorithm [11].

#### 2.1.2. System Calibration

When the sensors are attached to the body segments, their orientation relative to these segments is still unknown. Consequently, a calibration procedure is necessary to align the coordinate frame of each sensor with its corresponding body segment frame. The calibration procedures ensure that the sensors can be placed in any orientation on each body segment. The coordinate frames corresponding to the body segments are defined following ISB recommendations [26]. Moreover, the global reference frame is defined as follows: The *y*-axis is pointing upward, parallel to the direction of gravity; the *x*-axis is pointing in the direction of the horizontal component of the earth magnetic field vector; and the *z*-axis forms a right-handed Cartesian coordinate system.

The calibration procedure consists of two consecutive steps. The first step involves a five-second static calibration, during which a participant is required to stand still in a neutral upright pose. It is assumed that, during this period, the longitudinal axis of each body segment is aligned with the direction of gravity. The orientation of each sensor is rotated to a temporary frame associated with the sensor’s corresponding body segment. The temporary frame of each body segment is equal to the global reference frame during the static calibration. The average orientation of each sensor during the static calibration period is used to rotate the orientation of each sensor to the temporary frame:

(1) qTF−BSF−Bcal=1n∑i=1nqGFSF−Bcal,n1n∑i=1nqGFSF−Bcal,n ,

(2) qGFTF−Bt=qGFSF−Bt⊗qTF−BSF−Bcal* ,

where SF-B denotes the sensor frame corresponding to body segment B, GF denotes the global reference frame, TF-B denotes the temporary frame of body segment B, qTF−BSF−Bcal is the normalized average orientation of the TF-B relative to SF-B, subscript cal denotes the calibration period, n represents the number of data samples within the calibration period, subscript t denotes the time index of the orientation quaternion, ⊗ denotes a quaternion multiplication, and * denotes the complex conjugate of a quaternion [27].

**Figure 2:**
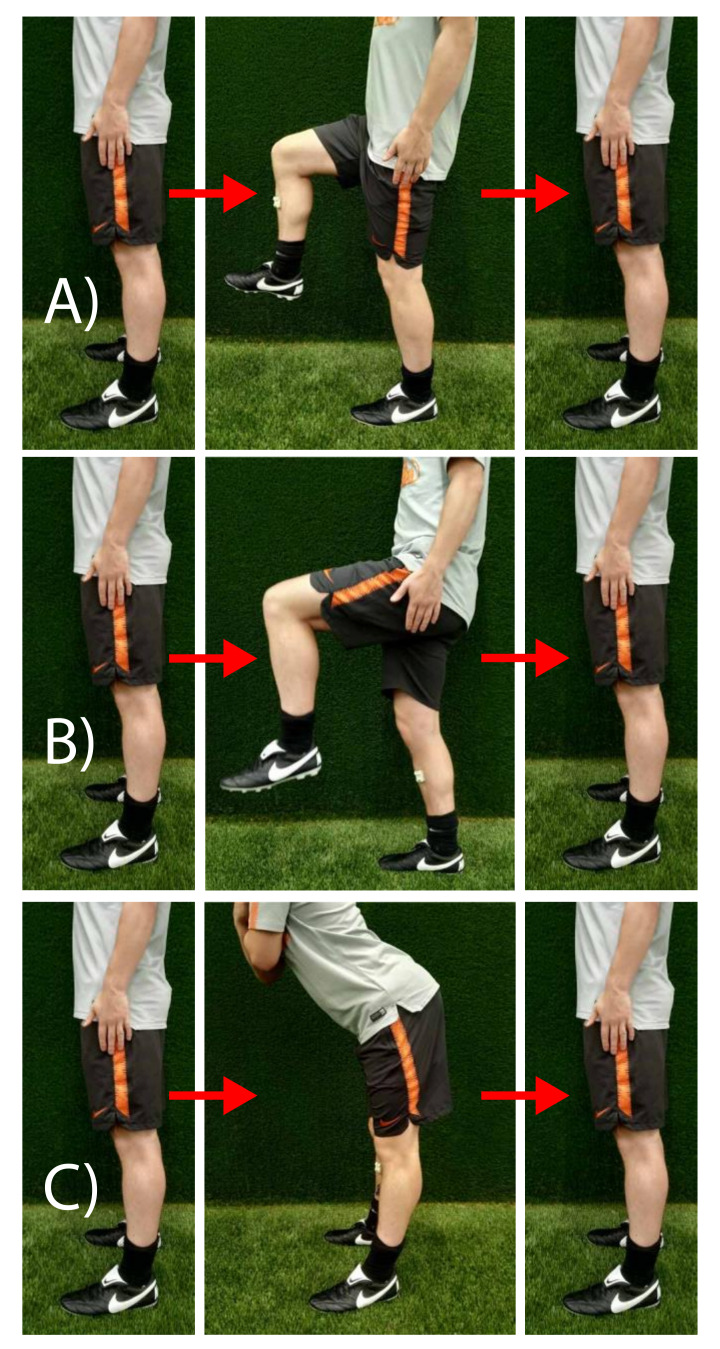
Calibration movements. Photographic representation of functional calibration procedures: (**A**) Rise of right leg, (**B**) rise of left leg, (**C**) bow forward.

The second step of the calibration procedure involves three functional calibration movements in order to determine the frontal axis of each segment. The calibration movements include a rise of the right upper leg (Figure 2A), a rise of the left upper leg (Figure 2B), and a bow forward of the trunk (Figure 2C). It is assumed that the rotation of the body segments in these movements are purely about the frontal axis (in the sagittal plane) of the body frames. When the calibration movements are executed correctly, that is, without rotations about the longitudinal axis, the calibration movements are about the sagittal and frontal axes of the temporary frames. Therefore, the rotation of the temporary frame, with respect to the body frame, about the longitudinal axes, is determined by the ratio between the magnitudes of rotation about the frontal and sagittal axes of the temporary frame. These magnitudes are obtained by simply integrating the angular rate measurements from the start of movement to the point where maximal rotation is reached:

(3) $$\theta_{x,cal}^{TF-B}=\frac{1}{n}\overset{n}{\underset{i=1}{\sum }}\omega_{x,cal}^{TF-B}\;,$$

(4) $$\theta_{z,cal}^{TF-B}=\frac{1}{n}\overset{n}{\underset{i=1}{\sum }}\omega_{z,cal}^{TF-B}\;,$$

where $\theta_{x,cal}^{TF-B}$ is the rotation of the temporary frame about its sagittal axis, $\theta_{z,cal}^{TF-B}$ is the rotation of the temporary frame about its frontal axis, and *n* is the number of data samples within the calibration movement. Since the duration of the calibration movement is very short (about one second), errors due to integration drift will be minimal. The rotation of the temporary frame, with respect to the body frame, can now be calculated as follows for the leg sensors:

(5) $$\phi_{cal,leg}=arctan\left(\frac{\theta_{z,cal}^{TF-B}}{\theta_{x,cal}^{TF-B}}\right)-\frac{\pi }{2}\;,$$

The relative orientation of the body frame, with respect to the temporary frame, is assumed to be equal for the shank and thigh sensor within each leg, because the knee acts as a pure hinge joint in the calibration movement. Since the calibration movement of pelvis (bow forward) is in the opposite direction, the following formula applies to obtain the relative angle between the body frame and the temporary frame of the pelvis:

(6) $$\phi_{cal,pelvis}=arctan\left(\frac{\theta_{z,cal}^{TF-B}}{\theta_{x,cal}^{TF-B}}\right)+\frac{\pi }{2}\;,$$

The rotation quaternions to rotate sensor data from the temporary frame to the body frame can then be constructed for each sensor:

(7) qTF−BBF−Bcal=cosϕcal2 0 sinϕcal2 0* ,

Consequently, this quaternion can be used to rotate sensor data from the temporary frame to the body frame:

(8) qGFBF−Bt=qGFTF−Bt⊗ qTF−BBF−Bcal ,

#### 2.1.3. Joint Kinematics

Once the system is calibrated, joint orientations can be extracted throughout a measurement period by calculating the orientation of a distal segment relative to its proximal segment:

(9) qBF−DBBF−PBjoint,t=qBF−DBGFt⊗qGFBF−PBt ,

where BF-PB denotes the body frame of the proximal body segment, and BF-DB denotes the body frame of the distal body segment. These joint orientation quaternions are then decomposed into ‘ZXY’ Euler angles in order to provide anatomically relevant joint angles [27]. The joint angular velocities are obtained by expressing the directly measured angular velocity of the distal segment in the coordinate frame of the proximal segment minus the angular velocity of the proximal segment expressed in the same coordinate frame:

(10) ωjoint,tBF−PB=qBF−DBBF−PBjoint,t⊗ωtBF−DB⊗ qBF−DBBF−PBjoint,t*−ωtBF−PB ,

### 2.2. Experimental Validation

#### 2.2.1. Participants

Eleven male amateur football players (age: 21.8 ± 3.2 years, height: 181 ± 6 cm, weight: 76.3 ± 11.4 kg), performing at least one training session and one match per week, participated in the validation study. The participants were free of injuries at the time of testing. All participants were informed about the experimental procedures by letter before testing and verbally on the day of testing. All participants gave their written consent. The study was conducted in accordance with the Declaration of Helsinki, and was approved by the local ethics committee of the VU Amsterdam (VCWE-2019-070R1). The study was conducted at the campus of the Royal Dutch Football Association in the spring of 2019. 

Before the testing session, participants performed 15-min football specific warm-up on an outdoor artificial turf football pitch. The warm-up comprised their usual pretraining warm-up and the possibility to stretch (to own preference). 

#### 2.2.2. Equipment

The participants were equipped with the inertial motion analysis system described above. Concurrent validity was assessed using eight optoelectronic motion cameras (Vicon V5 cameras, Vicon Motion Systems Ltd., Oxford, UK) sampling at 250 Hz as gold standard reference. The cameras were mounted at an approximate height of 2.3 m around the testing area. The optoelectronic motion analysis system was calibrated according to the manufacturer’s recommendations. Twenty retro-reflective markers were placed on the following anatomical landmarks; medial and lateral malleoli, the medial and lateral femoral epicondyles, the posterior and anterior superior iliac spines, the lateral and posterior side of each thigh half way the length hip to knee, and the lateral and anterior side of each shank halfway the length knee to ankle. The inertial-based motion analysis system was calibrated as described above.

#### 2.2.3. Protocol

**Figure 3:**
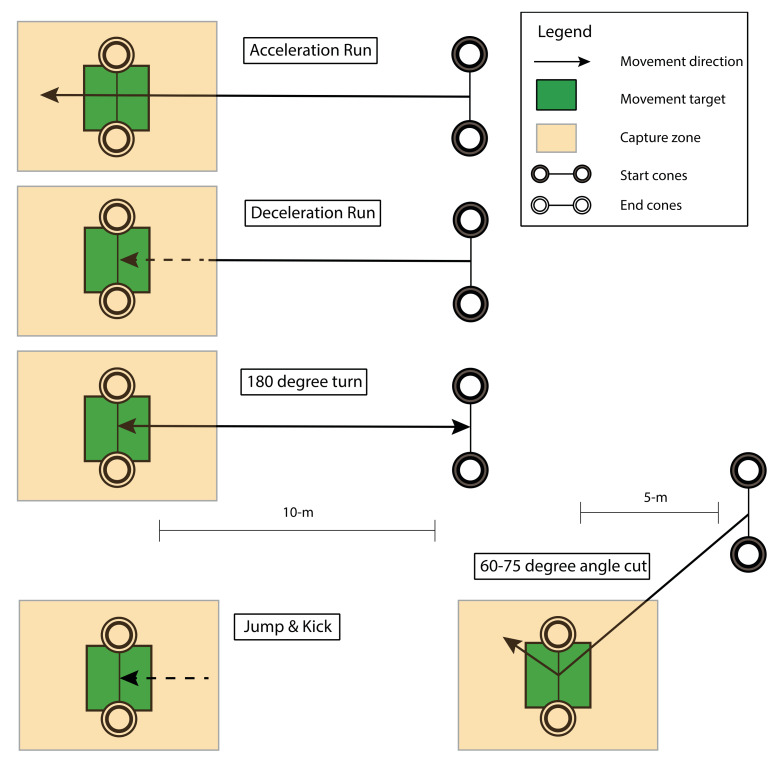
Football specific movements. Schematic representation of the performed football specific movements.

Six different football specific movements were performed in an indoor laboratory, equipped with artificial turf on the floor (width × length: 5 m × 15 m) and a 5 m × 5 m optoelectronic motion capture area. One side (5-m width) of the laboratory could be opened to the outside, such that participants were able to run from inside to the field outside the laboratory and to kick balls from the motion capture area onto the field. The chosen football specific movements (Figure 3) are frequently executed in training sessions and matches and included an acceleration run, a deceleration run, a run with a ±60/75^o^ side-step cut, a run with an 180^o^ turn, a jump, and a kick [22]. Each movement was performed at three different intensities: Low, medium, and maximal. Participants were instructed to perform the running tasks at about 50% of maximum effort (low intensity), about 80% of maximum effort (medium intensity), and at maximum effort (maximal intensity). The three intensities for the jumping task were defined as a jump from standstill at 80% of maximum effort (low intensity), a jump with a small run up at 80% of maximum effort (medium intensity), and a jump with a small run up performed with maximal effort (maximal intensity). All kicks were preceded by a few steps and the intensities were as follows: A short pass (low intensity), a long pass (medium intensity), and a maximum instep kick (maximal intensity). After each movement, the participants rested for about 10 s. Following the execution of a movement at the three intensities, the participants had approximately 60 s of rest. Four trials were recorded per movement and intensity. The trial with the best marker visibility in Vicon was selected for further processing. All movements were measured as one single continuous recording for the inertial motion analysis system, whereas, for the optoelectronic system, each movement was recorded separately.

#### 2.2.4. Data Processing

Raw marker trajectory data obtained by the optoelectronic motion analysis system were processed in Vicon Nexus (version 2.7.1, Vicon Motion Systems Ltd., Oxford, UK). Gaps in marker trajectories were filled using Nexus’ Woltring gap fill algorithm and rigid body gap fill. Thereafter, marker data was exported to Matlab (version 2018a for mac, The MathWorks, Inc., Natick, MA, USA). Marker trajectories were smoothed using a second-order Butterworth low-pass filter with a cutoff frequency of 12 Hz. This cutoff frequency was chosen based on visual inspection and is similar to what others used [19]. The location of the hip joint center was calculated according to the Vicon Plug-in-Gait model [28]. Furthermore, joint coordinate systems were constructed following ISB recommendations [29,30]. Three-dimensional kinematics of the knees and hips were directly calculated from these joint coordinate systems.

The data of the inertial motion analysis system was cut into smaller sections, with each section representing one movement executed at one intensity. Kinematic data obtained by the optoelectronic motion analysis system was up sampled to 500 Hz to match the sample frequency of the inertial data. Kinematic data of both motion capture systems were then synchronized by cross-correlating the angles of the knees and hips during the movements. To be able to compare the movement intensities of the acceleration runs with previous literature, mean running speeds were computed by differentiating the horizontal pelvis position obtained by the optoelectronic motion analysis system. Mean absolute joint angular velocities, from here on referred to as absolute angular velocities for readability, were obtained from the inertial data as a measure of movement intensity. Moreover, absolute and relative (percentage of gold standard optoelectronic motion analysis system) errors in absolute joint angular velocities were calculated.

#### 2.2.5. Statistical Analysis

The statistical analyses were performed in Matlab (version 2018a for Mac, The MathWorks, Inc., Natick, MA, USA) and SPSS (IBM SPSS Statistics for Mac, Version 26.0, IBM Corp., Armonk, NY, USA). The validity of the inertial-based system was assessed by computing the root mean square differences (RMSD) and coefficients of multiple correlation (CMC) [31] between knee and hip joint angles and angular velocities for each movement obtained by the optoelectronic system and inertial-based system. Opposed to traditional correlation coefficients, CMC also accounts for differences in offsets between the two systems. CMC values were interpreted as follows: Weak (<0.650); moderate (0.650–0.750); good (0.750–0.850); very good (0.850–0.950); excellent (>0.950) [32]. Differences in validity measures, absolute angular velocities, and errors in absolute and relative (percentage optoelectronic motion analysis system) angular velocities were assessed using repeated measures analysis of variance (ANOVA) with movement, movement intensity, joint (hip and knee), and body side (left and right) as factors (6 × 3 × 2 × 2). A Greenhouse-Geisser correction was applied if the assumption of sphericity was not met. Moreover, Bonferroni post-hoc tests were executed to determine differences between the different conditions. All tests were performed with a significance level of *p* < 0.05 and effect sizes were computed as partial ETA squared (η^2^). All data are presented as means ± standard deviations.

## 3. Results

### 3.1. Overall

The repeated measures ANOVAs did not reveal any significant effects of body side on any of the analyzed variables (RMSD angles: *p* = 0.128, η^2^ = 0.237, CMC angles; *p* = 0.197 η^2^ = 0.160, RMSD angular velocities; *p* = 0.540, η^2^ = 0.039, CMC angular velocities; *p* = 0.824, η^2^ = 0.005, absolute angular velocity; *p* = 0.618, η^2^ = 0.026). Therefore, we chose to only present the results of the left leg for clarity. Please refer to the Supplementary Materials for the results of the right leg. 

**Figure 4:**
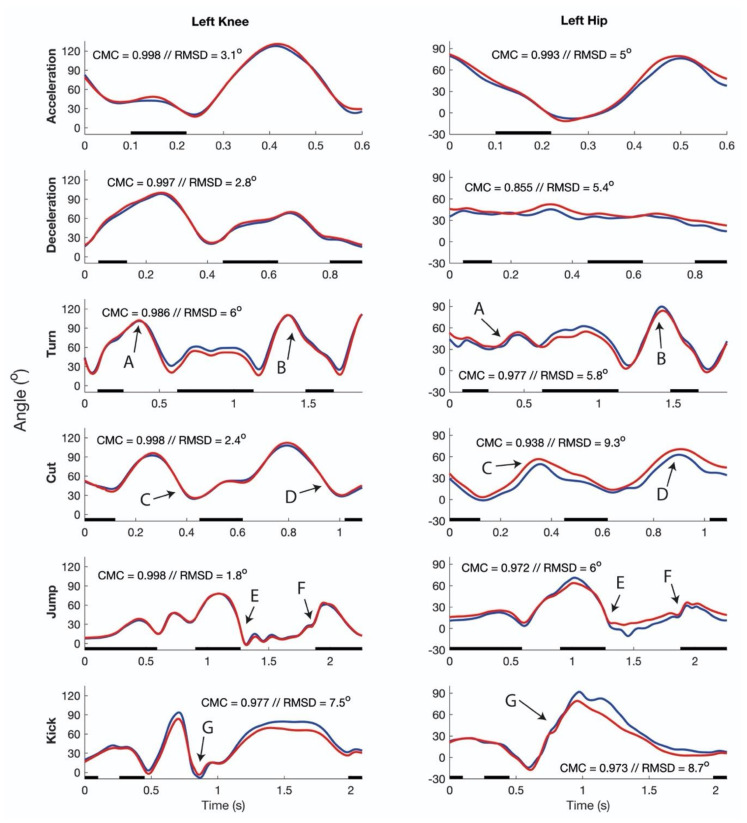
Typical results joint flexion/extension angles. Example of joint flexion/extension angles of the inertial-based motion analysis system (blue line) and the optoelectronic motion analysis system (red line) of all movements performed at maximum intensity by one participant. The black line indicates when the foot is in contact with the ground. For each joint and movement, the coefficient of multiple correlation (CMC) and root mean square difference (RMSD) is indicated in the respective graph. The arrows indicate events and have the following meanings: A = turn initiation, B = turn completion, C = cut initiation, D = cut completion, E = push-off, F = landing, G = moment of ball contact.

**Figure 5:**
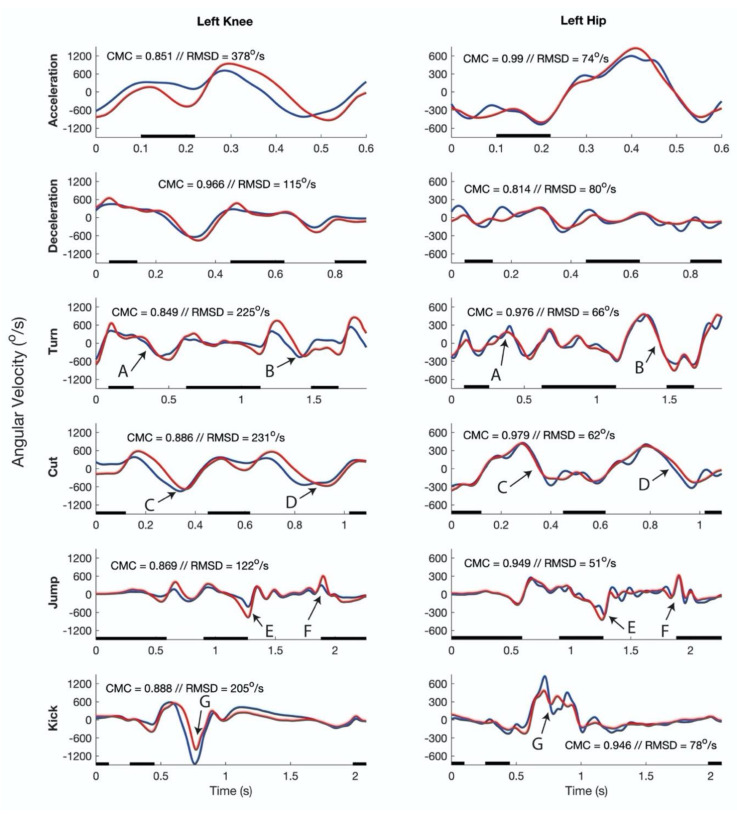
Typical results joint flexion/extension angular velocities. Example of joint flexion/extension angular velocities for the inertial-based motion analysis system (blue line) and the optoelectronic motion analysis system (red line) of all movements performed at maximum intensity by one participant. The black lines indicate when the foot is in contact with the ground. For each joint and movement, the coefficient of multiple correlation (CMC) and root mean square difference (RMSD) is indicated in the respective graph. The arrows indicate events and have the following meaning: A = turn initiation, B = turn completion, C = cut initiation, D = cut completion, E = push-off, F = landing, G = moment of ball contact.

Examples of the joint angles and angular velocities of all movements performed at maximal intensity by one of the participants are shown in Figure 4 and Figure 5, respectively. Mean RMSDs and CMC values of angles and angular velocities of all movements and movement intensities across the participants are shown in Table 1 and Table 2, respectively. Furthermore, the mean absolute angular velocities for each movement and intensity are presented in Table 2. There was a significant main effect of movement-type on RMSDs in joint angle (*p* = 0.015, η^2^ = 0.347), as well as on joint angular velocity RMSDs (*p* < 0.001, η^2^ = 0.960) and CMC’s (*p* = 0.001, η^2^ = 0.939). Moreover, significant effects of joint on joint angle CMCs (*p* < 0.001, η^2^ = 0.774), and on angular velocity RMSDs (*p* < 0.001, η^2^ = 0.989) and CMCs (*p* < 0.001, η^2^ = 0.779) were found. Higher CMCs were observed for the hip joint compared to the knee joint (*p* < 0.001), while respective RMSDs were lower (*p* < 0.001). Finally, movement intensity showed significant effects for RMSDs of joint angular velocities (*p* < 0.001, η^2^ = 0.982), but not on angular velocity CMCs (*p* = 0.077, η^2^ = 0.435).

### 3.2. Running Tasks

Mean running speeds during the acceleration runs were 3.5 ± 0.5 m/s, 5.1 ± 0.6 m/s, and 6.6 ± 0.3 m/s for the low, medium, and maximal intensity trials, respectively. Across the running tasks and intensities, mean CMC values of knee angles were excellent and ranged 0.979 to 0.993, whereas corresponding mean RMSDs ranged from 4.4° to 6.4°. Mean CMC values of hip angles were between 0.854 and 0.985, with corresponding mean RMSDs between 6.5° and 10.9°.

### 3.3. Jumping and Kicking

In the jumping task, mean RMSDs in knee angles ranged 3.7° to 4.2°, whereas mean RMSDs in hip angles ranged from 6.7° to 7.6°. Over all intensities, mean CMC values were excellent for the knees (0.990–0.994) and ranged from very good to excellent for the hips (0.943–0.952). Mean RMSDs in joint angular velocities were between 104°/s and 133°/s for the knees, and between 54°/s and 63°/s for the hips. Moreover, CMC values for joint angular velocities ranged 0.878 to 0.894 and 0.932 to 0.945 for the knees and hips, respectively. 

In the kicking tasks, mean RMSDs in joint angles were between 5.3° and 6.2° for the knees and between 7.4° and 8.3° for the hips. Mean corresponding CMC values were between 0.964 and 0.973 for the knees and between 0.957 and 0.971 for the hips, respectively. Mean CMC values for knee angular velocities ranged from 0.875 to 0.889 with mean RMSDs between 116°/s and 177°/s. For angular velocities of the hips, mean CMC values were ranged from 0.814 to 0.851, whereas mean RMSDs were between 78°/s and 121°/s.

### 3.4. Movement Intensity

Significant main effects of joint (*p* < 0.001, η^2^ = 0.975), intensity (*p* < 0.001, η^2^ = 0.935), and movement type (*p* < 0.001, η^2^ = 0.941) on absolute angular velocities were found. Absolute angular velocities were significantly higher for the knees compared to the hips (*p* < 0.001). All three movement intensities were significantly different from each other (*p* < 0.001), whereas absolute angular velocities were highest in the maximum intensity movements and lowest in the low intensity movements. All movements significantly differed from each other in terms of absolute angular velocity (*p* = 0.000–0.019), except the deceleration, which did not differ from the turn (*p* = 1.000) or the cut (*p* = 0.135).

Significant main effects of joint (*p* < 0.001, η^2^ = 0.767), intensity (*p* < 0.001, η^2^ = 0.659), and movement type (*p* = 0.021, η^2^ = 0.295) on absolute errors in absolute angular velocities were observed. However, when these errors were expressed as a percentage of the absolute angular velocities measured by the optoelectronic system, all main effects disappeared (joint: *p* = 0.708, η^2^ = 0.015, intensity: *p* = 0.600, η^2^ = 0.045), except for the effect of movement-type (*p* = 0.001, η^2^ = 0.552). Overall, relative errors of absolute angular velocity were 16.5% ± 1.3%.

## 4. Discussion

The main aim of present study was to introduce and evaluate a simple inertial-based motion analysis system by assessing its concurrent validity with a gold standard optoelectronic motion analysis system during a variety of football specific movements performed at a range of intensities. The secondary aim was to establish whether different movement intensities could be distinguished based on joint flexion/extension angular velocities. The results showed very good to excellent correlations for knee angles over the range of movements and intensities performed, whereas the correlations for hip angles were good to excellent. Good to excellent correlations were found for knee and hip angular velocities. Moreover, mean absolute angular velocities clearly differed between low, medium, and maximal movement intensities.

RMSDs in knee angles (4–6°) during running were slightly larger than what has previously been found with inertial based motion analysis during walking [13,17,33] and running [13] (knee angle: RMSD < 3.4°). However, all these studies used marker data obtained by optoelectronic cameras to construct the biomechanical model of the inertial sensor system. Clearly, this is not possible when the inertial based motion analysis system is used as stand alone. Consequently, the results of these previous studies do not translate directly to on-field use of inertial-based motion analysis systems. In the independent use of inertial-based motion analysis systems, the biomechanical model is generally constructed based on pre-known postures, functional calibration procedures, or, as in our study, on a combination of both, rather than on the position of anatomical bony landmarks obtained with an additional system [19,21,32,34]. This inevitably leads to differences between the biomechanical models of the inertial sensor and the optoelectronic system, which are dependent on marker placement for the optoelectronic system and on the execution of the calibration movements for the inertial based system. This effect probably contributed to the somewhat larger RMSDs in joint angles in comparison to these earlier studies [13,17,33]. RMSDs in joint angular velocities were not previously reported but are likely to have been partially determined by this same effect. Although RMSDs in joint angular velocities appear to be relatively high (Table 2), good to excellent correlations indicate that the course of angular velocity signals was similar between the optoelectronic motion analysis system and inertial based system.

The RMSDs and CMCs of joint angles found in the present study across a range of movements and intensities are comparable to results of walking, running, and kicking found in other studies that used independent biomechanical models [19,21,32,34]. However, the maximum running speed that has been reported in these studies was ~3.9m/s [21], whereas, in our study, participants had a mean running speeds of 3.5 ± 0.5 m/s, 5.1 ± 0.6 m/s, and 6.6 ± 0.3 m/s during the acceleration runs at low, medium, and maximal intensity, respectively. This indicates that our inertial-based motion analysis system still provides valid measures of joint angles at movements intensities that are considerably higher compared to previous research. 

However, we expected that a relatively high movement intensity would affect the inertial-based motion system’s accuracy in the two following ways. First, the accuracy of an inertial-based system relies upon the performance of the orientation estimation of each individual sensor. One of the assumptions of sensor fusion algorithms is that the measured direction of the acceleration is equal to the direction of the gravitational acceleration [11]. Therefore, the performance of orientation estimation is negatively influenced in presence of linear accelerations. As a result, the accuracy of inertial-based system may be lower during high intensity movements. Second, the presence of soft tissue between the bones and sensors on the skin can lead to soft tissue artefacts in sensor-derived segment orientations. Any deformation in soft tissue between a sensor and bone leads to errors in the estimated segment orientation. High-impact forces and strong muscle contractions associated with high-intensity movements may mean larger soft tissue deformations, which may result in a lower inertial-based system accuracy [35]. Unexpectedly, we did not find an effect of movement intensity on CMC or RMSD in joint angles. An explanation could be that our low intensity movements were performed at about 50% of maximal intensity, which may have already been high enough to introduce substantial soft tissue deformations. As a consequence, differences in soft tissue artefacts between the movement intensities may have been too small to result in significant effects of movement intensity on the validity measures.

To the best of our knowledge, no previous studies have reported statistics on the similarity between complete joint angular velocity signals obtained by optoelectronic and inertial-based motion analysis systems. We found lower CMCs of joint angular velocities compared to angles, indicating a lower accuracy of the inertial-based motion analysis system in determining angular velocities compared to angles. Movement intensity had a significant effect on RMSDs in angular velocities and on absolute errors in absolute angular velocities. However, there were no main effects of movement intensity on the corresponding CMCs and relative errors in absolute angular velocities. These results suggest that the absolute errors in angular velocity measurements are proportional to the magnitude of joint angular velocity. This proportionality, as well as the error margins, should be considered when interpreting joint angular velocities in daily football practice situations.

Movement intensity measures are frequently used in football practice to estimate training load [1,3]. However, previously available methods to measure movement intensity are unable to estimate intensity of movements with small global displacements that may still be accompanied by the high mechanical loading of ligaments, tendons, and muscles, such as kicking, jumping, and short sprints. The significant effect of movement intensity on absolute angular velocities in the present study shows that the intensity of all six investigated movements, including kicking and jumping, can be estimated by measuring joint angular velocities. Yet, it should be noted that the type of movement also largely determined the presented joint angular velocities. Therefore, the effects of movement intensity on angular velocities cannot be directly compared among different movements. Consequently, automatic movement recognition algorithms may have to be included in the estimations of training load.

The sensor setup used in present study does not allow football players to make slide tackles because the sensors may come off and/or bruise the player. In addition, equipping many individual players with five separate sensors is not feasible in daily practice. Therefore, we are currently working on integration of the sensors into tights or shorts, which have a centralized power supply placed at the lower back where it has less impact with the ground during slide tackles. This also makes it possible to further miniaturize the individual sensor units, since the battery is, by far, the largest component of the units used in the present study.

## 5. Conclusions

This paper introduced a simple method to obtain hip and knee joint kinematics using IMUs. The method showed good validity with a gold standard optoelectronic motion analysis system for six different football specific movements, even when these were performed at maximal intensity. These findings open the possibility to improve quantification of the player’s physical load during football. 

## Figures and Tables

**Table 1:** Results joint flexion/extension angles. Root mean square differences (RMSD) and coefficients of multiple correlation (CMC) between knee and hip flexion/extension angles of the left leg obtained by the optoelectronic and inertial-based motion analysis systems.

		Left Knee		Left Hip	
Movement-type	Intensity	RMSD (^o^)	CMC	RMSD (^o^)	CMC
Acceleration	low	4.7 ± 3.2	0.992 ± 0.009	7.4 ± 2.3	0.954 ± 0.047
	medium	5.1 ± 3.2	0.993 ± 0.009	7.5 ± 2.3	0.978 ± 0.014
	high	6.0 ± 2.7	0.990 ± 0.011	7.5 ± 2.4	0.985 ± 0.011
Deceleration	low	4.4 ± 2.7	0.991 ± 0.010	7.4 ± 2.1	0.906 ± 0.063
	medium	4.5 ± 3.1	0.992 ± 0.009	6.5 ± 3.6	0.888 ± 0.155
	high	5.1 ± 3.5	0.987 ± 0.018	7.9 ± 3.9	0.854 ± 0.184
Turn	low	5.1 ± 2.7	0.989 ± 0.012	8.5 ± 3.3	0.951 ± 0.034
	medium	6.2 ± 2.9	0.985 ± 0.015	10.4 ± 4.0	0.928 ± 0.052
	high	6.4 ± 3.2	0.979 ± 0.024	10.9 ± 5.9	0.913 ± 0.074
Cut	low	6.6 ± 3.5	0.982 ± 0.014	7.6 ± 3.3	0.953 ± 0.040
	medium	6.2 ± 2.4	0.987 ± 0.007	8.5 ± 2.8	0.951 ± 0.040
	high	6.4 ± 3.2	0.981 ± 0.018	8.6 ± 3.0	0.928 ± 0.052
Jump	low	3.7 ± 2.9	0.994 ± 0.007	7.8 ± 3.8	0.952 ± 0.045
	medium	3.9 ± 2.5	0.992 ± 0.010	7.4 ± 3.7	0.943 ± 0.054
	high	4.2 ± 2.6	0.990 ± 0.013	8.3 ± 4.8	0.948 ± 0.058
Kick	low	5.3 ± 4.9	0.970 ± 0.046	7.5 ± 3.7	0.957 ± 0.032
	medium	6.0 ± 5.7	0.964 ± 0.056	6.7 ± 2.6	0.971 ± 0.016
	high	6.2 ± 4.6	0.973 ± 0.028	7.6 ± 2.5	0.965 ± 0.042
Overall	All	5.3 ± 3.4	0.985 ± 0.022	8.0 ± 3.5	0.940 ± 0.075

**Table 2:** Results flexion/extension joint angular velocities. Mean absolute joint flexion/extension angular velocities of the left leg obtained by the inertial motion analysis system, and the root mean square differences (RMSD) and coefficients of multiple correlation (CMC) between knee and hip flexion/extension angular velocities obtained by the optoelectronic and inertial based motion analysis systems.

		Left Knee			Left Hip		
**Movement-Type**	**Intensity**	**Absolute Angular Velocity (^o^/s)**	**RMSD (^o^/s)**	**CMC**	**Absolute Angular Velocity (^o^/s)**	**RMSD (^o^/s)**	**CMC**
Acceleration	low	271 ± 40	193 ± 31	0.902 ± 0.035	147 ± 21	67 ± 19	0.964 ± 0.019
	medium	394 ± 52	278 ± 49	0.893 ± 0.028	225 ± 40	73 ± 17	0.981 ± 0.008
	high	449 ± 43	373 ± 48	0.858 ± 0.034	310 ± 48	93 ± 19	0.983 ± 0.005
Deceleration	low	214 ± 33	135 ± 25	0.935 ± 0.030	104 ± 14	58 ± 17	0.940 ± 0.037
	medium	264 ± 53	165 ± 41	0.935 ± 0.023	129 ± 27	82 ± 28	0.918 ± 0.064
	high	313 ± 101	174 ± 63	0.945 ± 0.026	140 ± 40	97 ± 32	0.882 ± 0.068
Turn	low	187 ± 27	158 ± 30	0.900 ± 0.038	120 ± 22	68 ± 12	0.942 ± 0.029
	medium	195 ± 45	180 ± 21	0.836 ± 0.211	138 ± 14	76 ± 14	0.947 ± 0.022
	high	205 ± 45	190 ± 39	0.897 ± 0.028	154 ± 29	94 ± 28	0.925 ± 0.050
Cut	low	253 ± 48	182 ± 27	0.896 ± 0.041	140 ± 22	63 ± 29	0.925 ± 0.042
	medium	316 ± 66	250 ± 42	0.873 ± 0.031	191 ± 31	85 ± 29	0.956 ± 0.028
	high	339 ± 55	253 ± 54	0.884 ± 0.042	202 ± 31	108 ± 36	0.958 ± 0.055
Jump	low	75 ± 15	104 ± 17	0.894 ± 0.018	75 ± 17	56 ± 16	0.932 ± 0.025
	medium	106 ± 18	119 ± 23	0.890 ± 0.030	88 ± 19	54 ± 10	0.939 ± 0.024
	high	112 ± 20	133 ± 15	0.878 ± 0.021	99 ± 12	63 ± 17	0.945 ± 0.038
Kick	low	124 ± 66	116 ± 65	0.889 ± 0.084	77 ± 50	78 ± 29	0.814 ± 0.158
	medium	155 ± 69	151 ± 79	0.887 ± 0.036	101 ± 61	106 ± 20	0.824 ± 0.105
	high	162 ± 63	177 ± 70	0.875 ± 0.039	116 ± 56	121 ± 22	0.851 ± 0.089
Overall	All	230 ± 113	185 ± 81	0.893 ± 0.064	142 ± 66	80 ± 29	0.925 ± 0.076

## Supplementary Materials

The following are available online at https://www.mdpi.com/1424-8220/20/9/2527/s1, Table S1: Results joint flexion/extension angles right leg, Table S2: Results flexion/extension joint angular velocities right leg, Software S1: Inertial based motion tracking example.
