# Supplementary material for: Inertial Sensor-Based Motion Tracking in Football with Movement Intensity Quantification
Source: Sensors (Basel). 2020 Apr 29;20(9):2527. doi: 10.3390/s20092527 (PMC7248913; doi:10.3390/s20092527)
Supplement: Supplementary file 1 [file sensors-20-02527-s001.zip › Supplementary materials/Supplementary materials tables.docx]

Table S1. **Results joint flexion/extension angles.** Root mean square differences (RMSD) and coefficients of multiple correlation (CMC) between knee and hip flexion/extension angles of the right leg obtained by the optoelectronic and inertial based motion analysis systems.

|  |  | Right Knee |  | Right Hip | |  |  |  |  |
| --- | --- | --- | --- | --- | --- | --- | --- | --- | --- |
| Movement - type | Intensity | RMSD (^o^) | CMC | RMSD (^o^) | CMC |  |  |  |  |
| Run | low | 6.2 ± 4.2 | 0.982 ± 0.021 | 7.3 ± 2.9 | 0.954 ± 0.037 |  |  |  |  |
|  | medium | 6.1 ± 3.7 | 0.991 ± 0.010 | 6.9 ± 1.9 | 0.980 ± 0.010 |  |  |  |  |
|  | high | 6.5 ± 4.2 | 0.989 ± 0.017 | 7.6 ± 3.2 | 0.982 ± 0.019 |  |  |  |  |
| Stop | low | 5.8 ± 3.3 | 0.984 ± 0.018 | 6.8 ± 2.1 | 0.920 ± 0.072 |  |  |  |  |
|  | medium | 6.4 ± 4.0 | 0.981 ± 0.020 | 6.4 ± 3.0 | 0.899 ± 0.103 |  |  |  |  |
|  | high | 6.3 ± 4.4 | 0.973 ± 0.036 | 6.9 ± 3.8 | 0.885 ± 0.138 |  |  |  |  |
| Turn | low | 7.5 ± 4.6 | 0.973 ± 0.029 | 7.7 ± 3.5 | 0.951 ± 0.046 |  |  |  |  |
|  | medium | 7.7 ± 5.0 | 0.974 ± 0.032 | 8.3 ± 3.8 | 0.955 ± 0.037 |  |  |  |  |
|  | high | 7.9 ± 5.1 | 0.965 ± 0.042 | 12.4 ± 6.9 | 0.838 ± 0.153 |  |  |  |  |
| Cut | low | 6.7 ± 4.7 | 0.976 ± 0.029 | 8.0 ± 2.8 | 0.943 ± 0.039 |  |  |  |  |
|  | medium | 7.2 ± 5.6 | 0.971 ± 0.043 | 8.6 ± 4.2 | 0.934 ± 0.062 |  |  |  |  |
|  | high | 8.2 ± 5.9 | 0.954 ± 0.074 | 9.2 ± 3.8 | 0.940 ± 0.044 |  |  |  |  |
| Jump | low | 6.3 ± 4.8 | 0.976 ± 0.027 | 7.3 ± 3.2 | 0.956 ± 0.040 |  |  |  |  |
|  | medium | 6.5 ± 4.4 | 0.973 ± 0.025 | 5.9 ± 1.9 | 0.965 ± 0.022 |  |  |  |  |
|  | high | 7.0 ± 4.9 | 0.973 ± 0.029 | 6.6 ± 1.8 | 0.968 ± 0.023 |  |  |  |  |
| Kick | low | 6.8 ± 4.2 | 0.950 ± 0.058 | 6.5 ± 2.9 | 0.951 ± 0.062 |  |  |  |  |
|  | medium | 7.0 ± 4.6 | 0.957 ± 0.045 | 5.9 ± 2.0 | 0.970 ± 0.017 |  |  |  |  |
|  | high | 6.3 ± 3.4 | 0.978 ± 0.018 | 6.2 ± 1.2 | 0.976 ± 0.015 |  |  |  |  |
| Overall | All | 6.8 ± 4.4 | 0.973 ± 0.036 | 7.5 ± 3.5 | 0.943 ± 0.073 |  |  |  |  |

Table S2. **Results flexion/extension joint angular velocities.** Mean absolute joint flexion/extension angular velocities of the right leg obtained by the inertial motion analysis system, and the root mean square differences (RMSD) and coefficients of multiple correlation (CMC) between knee and hip flexion/extension angular velocities obtained by the optoelectronic and inertial based motion analysis systems.

|  |  | Right Knee | | | Right Hip | | |  |  |  |  |
| --- | --- | --- | --- | --- | --- | --- | --- | --- | --- | --- | --- |
| Movement | Intensity | Absolute angular velocity (^o^/s) | RMSD (^o^/s) | CMC | Absolute angular velocity (^o^/s) | RMSD (^o^/s) | CMC |  |  |  |  |
| Run | low | 278 ± 72 | 190 ± 37 | 0.898 ± 0.059 | 154 ± 33 | 60 ± 13 | 0.968 ± 0.019 |  |  |  |  |
|  | medium | 374 ± 77 | 275 ± 50 | 0.883 ± 0.038 | 224 ± 44 | 74 ± 17 | 0.978 ± 0.010 |  |  |  |  |
|  | high | 454 ± 45 | 382 ± 47 | 0.856 ± 0.036 | 325 ± 35 | 86 ± 26 | 0.985 ± 0.008 |  |  |  |  |
| Stop | low | 207 ± 52 | 124 ± 25 | 0.929 ± 0.020 | 107 ± 14 | 55 ± 16 | 0.939 ± 0.040 |  |  |  |  |
|  | medium | 266 ± 70 | 146 ± 51 | 0.929 ± 0.033 | 123 ± 33 | 66 ± 16 | 0.913 ± 0.064 |  |  |  |  |
|  | high | 317 ± 95 | 150 ± 64 | 0.939 ± 0.040 | 144 ± 48 | 82 ± 36 | 0.865 ± 0.112 |  |  |  |  |
| Turn | low | 191 ± 28 | 149 ± 13 | 0.909 ± 0.025 | 119 ± 16 | 67 ± 22 | 0.940 ± 0.034 |  |  |  |  |
|  | medium | 204 ± 36 | 192 ± 52 | 0.878 ± 0.057 | 140 ± 20 | 77 ± 32 | 0.945 ± 0.042 |  |  |  |  |
|  | high | 221 ± 58 | 201 ± 25 | 0.870 ± 0.088 | 159 ± 33 | 101 ± 30 | 0.921 ± 0.054 |  |  |  |  |
| Cut | low | 256 ± 48 | 171 ± 37 | 0.904 ± 0.049 | 131 ± 23 | 71 ± 30 | 0.945 ± 0.037 |  |  |  |  |
|  | medium | 295 ± 38 | 213 ± 47 | 0.879 ± 0.041 | 173 ± 40 | 87 ± 39 | 0.943 ± 0.043 |  |  |  |  |
|  | high | 328 ± 45 | 253 ± 65 | 0.875 ± 0.052 | 204 ± 54 | 109 ± 37 | 0.928 ± 0.076 |  |  |  |  |
| Jump | low | 72 ± 15 | 100 ± 22 | 0.881 ± 0.024 | 75 ± 16 | 51 ± 14 | 0.936 ± 0.020 |  |  |  |  |
|  | medium | 105 ± 18 | 109 ± 24 | 0.880 ± 0.034 | 86 ± 17 | 47 ± 9 | 0.944 ± 0.030 |  |  |  |  |
|  | high | 109 ± 27 | 126 ± 32 | 0.867 ± 0.029 | 97 ± 18 | 55 ± 13 | 0.940 ± 0.033 |  |  |  |  |
| Kick | low | 147 ± 75 | 122 ± 60 | 0.861 ± 0.078 | 95 ± 51 | 65 ± 22 | 0.835 ± 0.233 |  |  |  |  |
|  | medium | 182 ± 79 | 136 ± 57 | 0.904 ± 0.025 | 110 ± 47 | 89 ± 19 | 0.838 ± 0.095 |  |  |  |  |
|  | high | 218 ± 112 | 185 ± 75 | 0.887 ± 0.027 | 151 ± 72 | 113 ± 27 | 0.876 ± 0.063 |  |  |  |  |
| Overall | All | 235 ± 112 | 181 ± 82 | 0.891± 0.050 | 145 ± 68 | 76 ± 31 | 0.924 ± 0.084 |  |  |  |  |
